# Decarbonatization of Energy Sector by CO_2_ Sequestration in Waste Incineration Fly Ash and Its Utilization as Raw Material for Alkali Activation

**DOI:** 10.3390/ma16186094

**Published:** 2023-09-06

**Authors:** Jakub Mokrzycki, Paweł Baran, Magdalena Gazda-Grzywacz, Jakub Bator, Wojciech Wróbel, Katarzyna Zarębska

**Affiliations:** Department of Coal Chemistry and Environmental Sciences, Faculty of Energy and Fuels, AGH University of Science and Technology, Mickiewicza 30 Av., 30-059 Cracow, Poland; jmokrzycki@agh.edu.pl (J.M.); baranp@agh.edu.pl (P.B.); magdago@agh.edu.pl (M.G.-G.); bator@agh.edu.pl (J.B.); wwrobel@khk.krakow.pl (W.W.)

**Keywords:** fly ash, mineral carbonation, alkali activation, waste material

## Abstract

In this study, municipal solid waste incineration (MSWI) fly ash was subjected to mineral carbonation with the aim of investigating CO_2_ sequestration in waste material. The conducted study follows the trend of searching for alternatives to natural mineral materials with the ability to sequestrate CO_2_. The mineral carbonation of MSWI fly ash allowed for the storage of up to 0.25 mmol CO_2_ g^−1^. Next, both carbonated and uncarbonated MSWI fly ashes were activated using an alkaline activation method by means of two different activation agents, namely potassium hydroxide and potassium silicate or sodium hydroxide and sodium silicate. Mineral carbonation caused a drop in the compressive strength of alkali-activated materials, probably due to the formation of sodium and/or potassium carbonates. The maximum compressive strength obtained was 3.93 MPa after 28 days for uncarbonated fly ash activated using 8 mol dm^−3^ KOH and potassium hydroxide (ratio 3:1). The relative ratio of hydroxide:silicate also influenced the mechanical properties of the materials. Both carbonated and uncarbonated fly ashes, as well as their alkali-activated derivatives, were characterized in detail by means of XRD, XRF, and FTIR. Both uncarbonated and carbonated fly ashes were subjected to TG analysis. The obtained results have proved the importance of further research in terms of high-calcium fly ash (HCFA) utilization.

## 1. Introduction

Fly ash (FA) is a solid by-product obtained during solid fuel combustion in power plants, i.e., coal, lignite, and biomass, and from municipal waste incineration. Annual global production of FA is about 800 Mt, and to a greater extent, it is being landfilled, which is highly space-consuming [1]. Constantly growing demand for energy production in upcoming years will result in an increase in its quantity [2]. Its negative impact on the environment is reflected by the potential risk of heavy metals and/or radionuclides leaching into the soil, groundwaters, and rivers [3]. The share of the abovementioned contaminants is strictly dependent upon the source and type of used solid fuel [4]. Another problem is the emission of particulate matter (PM), which is known due to its potential risk to human health. In recent years, the global utilization of fly ashes has significantly increased. It was reported that only about 30% of global fly ash produced is being reused, from which about 65% is being utilized in the cement industry [5]. However, there is a constant need for other potential utilization methods of FAs [6]. Great attention is being paid nowadays to so-called class C fly ashes or class F fly ashes containing extra calcium, as a high calcium content was reported to lower the mechanical properties of FAs’ alkali-activated derivatives [7]. Some recent findings underline the utilization of fly ashes as additives to cement [8] and raw materials for porous materials syntheses such as zeolites [9,10,11], mesoporous silica [12], metal-organic frameworks (MOFs) [13], and alkali-activated materials [14,15].

Alkali activation allows for the immobilization of hazardous wastes into solid blocks. The most common alkali-activated materials are geopolymers. Geopolymers are a class of inorganic compounds obtained from various aluminosilicate raw materials during their alkaline activation using KOH or NaOH solutions as alkaline agents [16]. As a result of polycondensation (geopolymerization), an amorphous, three-dimensional network is formed [17]. The mechanism of geopolymerization is rather complex and is constantly under development. However, some main stages involve the (i) initial transportation of hydroxyl ions to the surface of aluminosilicate raw material, (ii) dissolution of aluminosilicate raw material and its hydrolysis by activating agent-forming alumina and silica tetrahedron units, (iii) oligomerization of as-formed monomers forming an initial geopolymeric gel phase, (iv) polycondensation of the geopolymeric gel to a geopolymer, and (v) hardening of the geopolymer [18]. As the liquid phase is a complex mixture, these steps, in fact, occur simultaneously and can affect one another [19]. Much research can be found considering the effect of operating conditions on geopolymer synthesis and properties, namely, the type of various aluminosilicate raw materials (fly ash [14], blast furnace slag [20], metakaolin [21]), Si/Al ratio of raw material (and share of reactive silica and alumina) [22], alkaline activating agent concentration and dose [23], solid to liquid ratio [24], temperature and curing time [25]. In addition, the silicate-to-hydroxide ratio has been underlined to affect the geopolymerization process (shorter gelation time for higher silicate/hydroxide ratio) [26].

As a result of the selected synthesis parameters, the physicochemical properties of geopolymers can be controlled. Their most important features are excellent thermal resistance, fire resistance, and strong acid resistance. Some other properties involve heavy metals immobilization and acoustic absorption. One of the key features is the opportunity to synthesize geopolymers from waste materials [27].

However, geopolymers require a suitable Si/Al ratio of raw materials, with simultaneous low content of free CaO (<1.5%) to obtain materials characterized by sufficient mechanical properties, i.e., compressive strength (>40 MPa). For this reason, some groups of raw materials, especially those obtained during waste incineration, are considered difficult for further treatment. Their chemical composition involves high contents of CaO, SiO_2_, Cl (chlorides), K_2_O, Na_2_O, SO_3_, and heavy metal oxides. There are, however, limited data considering the effect of various alkali activation treatments on the mechanical properties of solid products, which is reported to not exceed 10 MPa (compressive strength) [28,29]. For example, in the work of Beaino et al. [30], the addition of waste biomass (rice husk) to high-calcium fly ash did not significantly improve the compressive strength of the material, which was reported to be <3 MPa. On the other hand, due to the high content of CaO, MSWI fly ashes can be considered promising materials for CO_2_ sequestration via mineral carbonation. Such treatment has already been reported to minimize the cost of CO_2_ storage in power plants, where mineral carbonation can be conducted in situ without the need for CO_2_ or fly ash transportation [31]. In fact, the global production of CO_2_ as waste from the energy sector reached above 43 Gt in 2019, and its amount has increased annually. Aiming to achieve carbon neutrality, it is necessary to minimize total CO_2_ emissions to the atmosphere. Hence, mineral carbonation can be considered an efficient and economically friendly method for carbon capture, utilization, and storage [32]. The use of high-calcium fly ash could provide an interesting raw material source for the mineral carbonation process. If used as a natural feedstock, its cost in the total process would be about 50% [33]. Assuming a sequestration of 1 Gt CO_2_ year^−1^, this would require the extraction of more than 2.5 Gt year^−1^ of natural mineral feedstock, implying the need to mine almost 100 kt day^−1^. Replacing this raw material with high-calcium fly ash or another industrial solid waste would be highly justifiable in this situation while lowering OPEX costs. CO_2_ sequestration has been widely investigated over various materials, including (i) natural minerals such as forsterite (Mg_2_SiO_4_), serpentinite (Mg_3_Si_2_O_5_(OH)_4_), or wollastonite (CaSiO_3_) and (ii) industrial solid wastes such as fly ash, blast furnace slag, and steel slag [34]. Mineral carbonation has been evaluated under various conditions; however, in work [35], the authors claimed that lower temperatures (ambient) and higher gas pressures can aid the mineral carbonation process.

The present study had a dual aim. First, fly ash from a municipal waste incineration plant was carbonated using CO_2_ under saturated steam pressure for 48 h at 25 °C. The adsorption capacity of fly ash was determined by mass difference after the process. Next, both raw fly ash and carbonized fly ash were subjected to an alkaline activation using (i) 8 mol dm^−3^ KOH and potassium silicate or (ii) 8 mol dm^−3^ NaOH and sodium silicate at two different relative hydroxide:silicate ratios of 3:1 and 1:3. The obtained alkali-activated materials from both carbonized and uncarbonized fly ashes were investigated for their mechanical properties using a compressive strength test after 7 and 28 days. Hence, in this paper, we have presented the effect of fly ash mineral carbonation, as well as the effect of selected alkaline activation agents and their relative ratio on the mechanical properties of the investigated materials. To the best of the authors’ knowledge, there are no reports on the effect of mineral carbonation on the mechanical properties of alkali-activated materials. To gain a better understanding of their physicochemical properties, the materials were characterized in detail by means of XRD, XRF, and FTIR. Additionally, fly ashes, both prior to and after mineral carbonation, were evaluated by means of TG analysis. In addition, their free CaO content was examined.

## 2. Materials and Methods

### 2.1. Fly Ash

In this study, fly ash from the selected municipal solid waste incineration plant was used and denoted as FGT (obtained from the flue gas treatment system). Samples were provided by the producer as homogenized powder. Fly ash after flue gas treatment (FGT) system is much finer and contains a higher content of heavy metals. This poses a major problem for waste incinerators; as such, the waste is treated as hazardous waste. Hence, we evaluated the chemical composition of our samples to show the potential risks that must be underlined when working with such a material. The chemical composition of municipal solid waste incineration fly ashes, which are complicated matrices, can differ significantly depending on the used feedstock for waste combustion. The material used for this research was provided by the selected municipal solid waste incineration company and was unified by the producer, delivered in a 10 kg box as a homogenized powder. Proper collection of samples was also crucial and allowed us to unify the MSWI fly ash while preparing the alkali-activated materials.

#### 2.1.1. Fly Ash Treatment with CO_2_

**Scheme 1:**
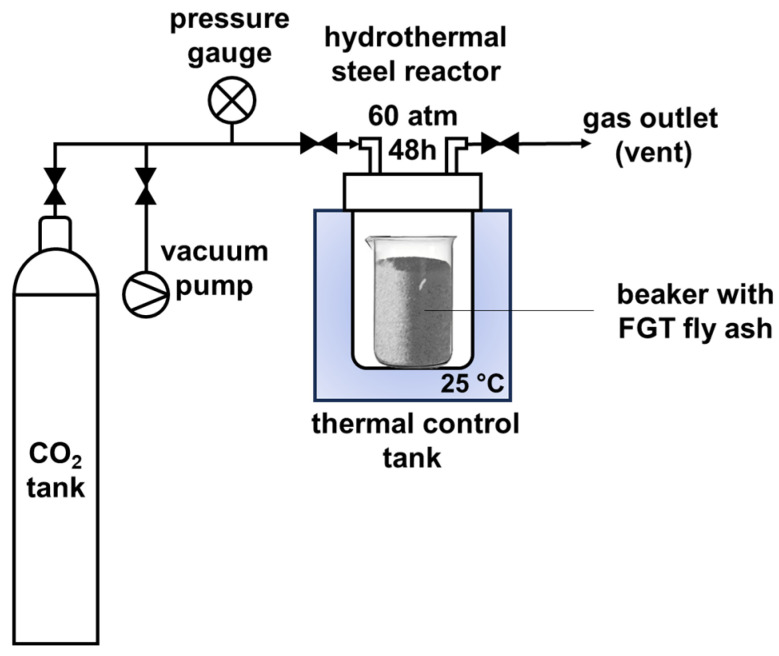
Mineral carbonation of MSWI fly ash.

To investigate the effect of fly ash exposure to CO_2_, FGT fly ash sample of 400 ± 0.010 g was weighed into a flask and placed in a stainless steel reactor. Mineral carbonation was conducted using a static method. Briefly, air was removed using a vacuum pump, and then the CO_2_ was supplied from the bottle to obtain the saturated steam pressure of CO_2_ (about 60 bar) within the reactor. The reactor was autoclaved to maintain constant temperature of 25 ± 2 °C. Mineral carbonation was conducted for 48 h. The selection of the abovementioned parameters was due to observations from recent studies [36,37]. Moreover, such conditions allowed us to lower the cost of the process. Next, the fly ash sample was collected and weighed to determine the CO_2_ adsorption capacity by mass difference. Material was stored for further investigations. The obtained fly ash was denoted as CO_2_FGT. The mineral carbonation process is demonstrated in Scheme 1.

#### 2.1.2. Fly Ash Alkali Activation

**Scheme 2:**
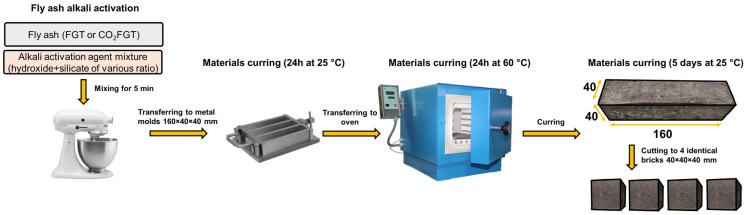
Fly ash alkali activation procedure.

Fly ashes were subjected to an alkaline activation. Briefly, 250 g of fly ash (FGT or CO_2_FGT) was weighed and mixed with activation agents composed from 8 mol dm^−3^ KOH solution or 8 mol dm^−3^ NaOH solution (Avantor, Gliwice, Poland) and potassium silicate or sodium silicate (Biomus, Lublin, Poland), respectively. Fly ash was homogenized in mixer for 1 min, while activation agents were mixed together in a beaker. Next, activation agent mixture was poured into the mixer and mixed together for another 4 min. To obtain a dough-like consistency, additional distilled water was added to the mixture if needed after 4 min (from 40 to 70 cm^3^). Two different hydroxide:silicate ratios of 3:1 and 1:3 were investigated, maintaining constant solid:activation agent ratio of 0.68. After 5 min of mixing, the mixture was transferred into metal molds of 40 × 40 × 160 mm^3^ and left for 24 h at room temperature. After 24 h, the molds were placed in an electric heated oven at 60 ± 2 °C for 24 h for materials curing. After thermal treatment, the samples were stored for further analyses. The samples were denoted as given in Table 1. Schematic illustration of alkali activation is presented in Scheme 2.

### 2.2. Methods of Materials Characterization

#### 2.2.1. X-ray Diffraction (XRD)

X-ray diffraction (XRD) was employed to investigate the phase composition of investigated fly ashes and obtained alkali-activated materials. Measurement was conducted using PANalytical Empyrean diffractometer (Malvern Panalytical, Malvern, UK). The radiation source was CuKα λ = 1.5406 Å, and the 2θ range varied from 3° to 90°, with a 0.013° step.

#### 2.2.2. X-ray Fluorescence (XRF)

X-ray fluorescence (XRF) was used to investigate the composition of investigated fly ashes and alkali-activated materials. ARL QUANT’X spectrometer (Thermo Scientific, Waltham, MA, USA) was used for analyses.

#### 2.2.3. Fourier Transform Infrared Spectroscopy (FT-IR)

Attenuated Total Reflection Fourier Transform Infrared Spectroscopy (ATR-FTIR) measurements were performed using spectrometer (Spotlight 400, Perkin Elmer, Waltham, MA, USA) apparatus performing in transmittance mode, and the wavenumber range was from 4000 to 550 cm^−1^.

#### 2.2.4. Free CaO Content in Fly Ashes

Free calcium oxide (CaO) (*W*_CaO_) content was determined according to PN-EN 451-1:2017 [38]. In this method, 1 ± 0.001 g of fly ash was weighed in a 0.25 dm^3^ flask and refluxed with mixture containing 0.012 dm^3^ of ethyl acetoacetate (99% + pure, Acros Organics, Geel, Belgium) and 0.080 dm^3^ of butan-2-ol for 3 h. Next, the mixture was centrifuged and filtrated, and the residue was washed with 0.050 dm^3^ of propan-2-ol. The filtrate was titrated using 0.1 mol dm^−3^ HCl (Avantor, Gliwice, Poland) and bromophenol blue as indicator (yellow color of the solution indicates the end of titration). *W*_CaO_ content was calculated using Equation (1):

(1) $$W_{CaO}=\frac{28.04\times 0.1\times V}{m}\times 100\%$$

where 28.04 (g mol^−1^) is ½ mass of CaO; 0.1 (mol dm^−3^) is the concentration of HCl solution, *V* (dm^3^) is the volume of HCl solution used for titration, and *m* (g) is the mass of fly ash used for analysis.

#### 2.2.5. Thermogravimetric Analysis (TGA) of Fly Ashes

TGA was performed using Q5000IR apparatus (TA Instruments, New Castle, DE, USA). About 20 mg of sample was weighed and equilibrated in He flow of 25 dm^3^ min^−1^ for 5 min at 25 °C. Next, the sample was heated to 800 °C in a flow of synthetic air of 0.1 dm^3^ min^−1^ with temperature ramp of 10 °C min^−1^.

#### 2.2.6. Compressive Strength of Alkali-Activated Materials

The compressive strength was conducted using an automatic press (Multiserw, Brzeznica, Poland) following the standard method PN-EN196-1:2016-07 [39], which is used to define the mechanical properties of cement. Cubic bricks (40 × 40 × 40 mm^3^) were used for compressive strength determination at 7 and 28 days. The result is given as a mean value from a duplicate experiment.

## 3. Results and Discussion

### 3.1. CO_2_ Sequestration

Parent FGT fly ash was subjected to mineral carbonation in a stainless steel reactor under saturated CO_2_ steam pressure. The obtained CO_2_FGT fly ash was weighed after 48 h of CO_2_ exposure. The mass increased by about 1.25% (0.25 mmol CO_2_ g^−1^), implying direct adsorption of CO_2_ within the material via physical and/or chemical adsorption. Comparable values have also been reported in the literature. In work [40], the authors found that fly ash can store up to 1.36 mmol CO_2_ g^−1^ (60 g CO_2_ kg^−1^). On the other hand, it was reported by [41] that the efficiency of mineral carbonation of fly ash was 0.30 mmol CO_2_ g^−1^. In the research of [42], it was found that mineral carbonation of Ca(OH)_2_ within fly ash requires moisture to accelerate sequestration due to better availability of adsorption active centers. Such phenomenon was also well demonstrated in the research of Miao et al. [43], where it was found that conducting mineral carbonation in a liquid phase allows to yield a higher CO_2_ sequestration rate of up to 2.91 mmol CO_2_ g^−1^ (128 g CO_2_ kg^−1^). It should be noted, however, that a solid–liquid phase fly ash treatment requires further separation steps, which increases the costs of the sequestration. Overall, mineral carbonation of MSWI fly ash can be considered an efficient method for carbon sequestration.

### 3.2. Materials Characterization

**Figure 1:**
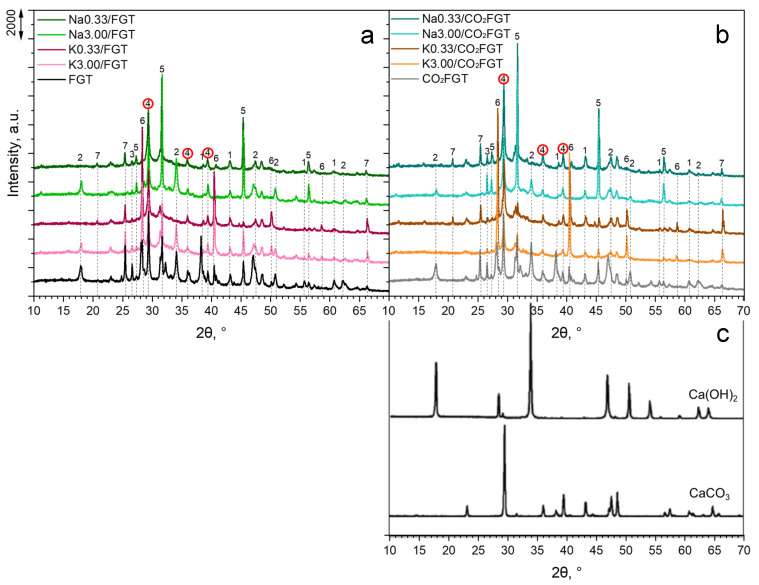
XRD patterns of FGT (**a**) and CO_2_FGT (**b**) fly ashes and their alkali-activated derivatives; (**c**) Ca(OH)_2_ and CaCO_3_ XRD patterns. 1—CaClOH, 2—Ca(OH)_2_, 3—CaSO_4_, 4—CaCO_3_, 5—NaCl, 6—KCl, and 7—SiO_2_.

The phase composition of fly ashes and obtained alkali-activated materials was investigated by means of XRD and is presented in Figure 1a,b. In Figure 1c, the XRD patterns of Ca(OH)_2_ and CaCO_3_ are presented for comparison. Fly ashes were composed of chloride compounds, including KCl and NaCl, and calcium-containing compounds such as CaCO_3_, CaSO_4_, and Ca(OH)_2_ [44]. The low content of the SiO_2_ phase was also confirmed within the fly ashes. The occurrence of the CaClOH phase might be related to the absorption of HCl and was also reported by Wu et al. [45]. It can be observed that the addition of sodium- or potassium-containing activation agents resulted in a significant increase in NaCl or KCl phases within the corresponding alkali-activated materials. It was also reported in the literature that even the low addition of an alkaline activator can accelerate the formation of NaCl, KCl, CaCl_2_, and MgCl_2_ [46,47,48]. Calcium carbonate (No. 4 in Figure 1 marked with a red circle) was more dominant within the CO_2_FGT fly ash and its alkali-activated derivatives and was a result of FGT fly ash mineral carbonation. It was also reflected by a decrease in the Ca(OH)_2_ peaks, for which the intensities were significantly lower in CO_2_FGT materials when compared with FGT. The following reaction (Equation (2)) might have occurred during the mineral carbonation of FGT fly ash.

Ca(OH)_2_ + CO_2_ = CaCO_3_ + H_2_O (2)

**Figure 2:**
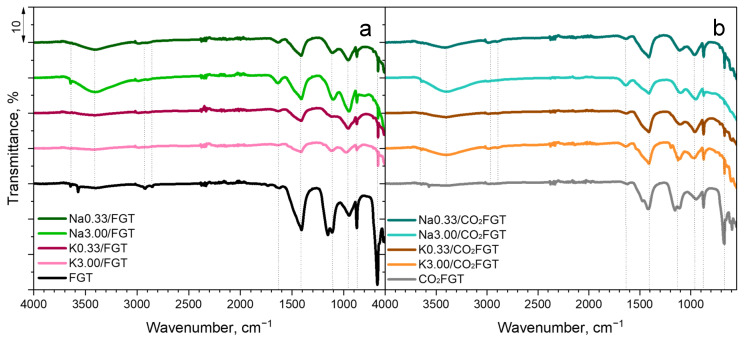
ATR-FTIR spectra of FGT (**a**) and CO_2_FGT (**b**) fly ashes and their alkali-activated derivatives.

In Figure 2, the FTIR spectra of investigated materials are presented. The raw data were also provided in Table S1. The main peaks involved 3653 cm^−1^ stretching vibrations from O–H groups within Ca(OH)_2_ and were the most dominant in the FGT fly ash; its intensity decreased in CO_2_FGT as a result of mineral carbonation. The broad peaks at about 3400 cm^−1^ and at 1639 cm^−1^ were identified as O–H stretching and bending vibrations from water molecules, respectively, and occurred the most in sodium-alkali-activated fly ashes, both carbonized and uncarbonized [49]. Two peaks in the range from 2929 to 2851 cm^−1^ were assigned to C–H stretching vibrations of the aliphatic chain and might be related to unburned carbonaceous material within the FGT fly ash. Peaks at 1110 and 953 cm^−1^ were identified as Si–O–Si and/or Si–O–Al bond vibrations and are typical for silica-containing materials. Peaks at 1430, 872, and 713 cm^−1^ were identified as calcium carbonate [50]. It can be clearly seen that the intensities of certain peaks differ between FGT and CO_2_FGT fly ashes. Hence, it can be stated that mineral carbonation affected the parent FGT fly ash, forming new bindings between fly ash and CO_2_. Treatment of both FGT and CO_2_FGT fly ashes with sodium activation agents resulted in the appearance of O–H bindings, which were more dominant when compared with treatment using potassium activation agents.

To gain a better understanding of the chemical composition of the investigated samples, X-ray fluorescence was employed. The summarized results are presented in Table 2. It can be seen that the main constituent of both FGT and CO_2_FGT fly ashes, as well as their alkali-activated derivatives, was (CaO)—about 52%. Such high content of CaO within the materials can significantly affect the alkali-activated materials obtained during activation and their mechanical properties [7]. Moreover, according to the European Union standard (EU 450-1:2012 [51]), high-calcium fly ash (HCFA) can be utilized as an additive to cements only when their free CaO content is <1.5%. For the investigated fly ashes, the *W*_CaO_ value was as high as 16.25 ± 0.20 and 15.41 ± 0.17% for FGT and CO_2_FGT, respectively. For such reason, their utilization as additives to cement would not be applicable from the point of view of the standard. However, it should be noted that mineral carbonation lowered the share of free CaO within the materials by 5%. Chlorine (Cl), along with sulfur (given as SO_3_), were the second most dominant components of fly ashes and the resulting alkali-activated materials. The significantly lower content of free CaO when compared with the total CaO content might be a result of CaSO_4_, CaCO_3_, or CaCl_2_ formation, the presence of which was also proved in the powder X-ray diffraction (Figure 1a,b). Both sodium and potassium alkali activation agents caused a decrease in the contents of SO_3_ and Cl. It was found that activation using an excess amount of silicate (ratio 0.33) resulted in a greater amount of potassium or sodium within the samples. Moreover, the content of SiO_2_ was higher when the hydroxide:silicate ratio was 0.33. Regardless of the hydroxide:silicate ratio, the content of SiO_2_ increased nearly 5-fold within the alkali-activated materials. Alkali activation appeared to have a rather negligible effect on the share of other components of the materials. Trace amounts of heavy metal oxides, including PbO, As_2_O_3_, and TiO_2_, were also identified; however, their share was <1%. Such pollutants are often identified within the waste incineration fly ashes. Additionally, alkali activation can serve as a method for the stabilization of such pollutants within solid materials [36,52,53]. Only trace amounts of Al were detected (<0.05%); for such reason, the results were not provided in Table 2.

**Figure 3:**
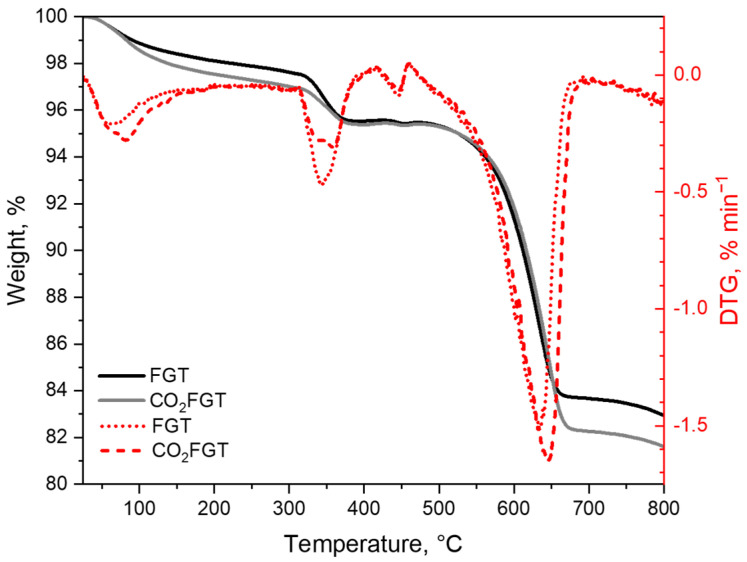
TG and DTG analysis of FGT and CO_2_FGT fly ashes.

TG analysis was conducted for uncarbonated (FGT) and carbonated (CO_2_FGT) fly ashes with the aim of investigating the effect of mineral carbonation. The results are presented in Figure 3. It can be seen that for both fly ashes, four stages of mass loss were detected: from 30 to 150, from 315 to 410, from 420 to 460, and from 420 to 690 °C. Initial mass loss was attributed to moisture release from the samples and was slightly greater for CO_2_FGT fly ash. It might also be related to the release of physically bonded CO_2_ from the fly ash surface. The second peak was assigned to dehydration occurring for Ca(OH)_2_, which was present within the material and was more dominant for FGT than for CO_2_FGT and was related to the formation of CaCO_3_ during mineral carbonation. The peak at about 440 °C might be related to the decomposition of CaClOH; however, it was reported to occur at slightly higher temperatures [54]. The most intensive peak, which occurred at about 640 °C, could be related to the decomposition of CaCO_3_ and was more dominant for carbonated fly ash, which proves the sequestration abilities of FGT fly ash [55,56].

### 3.3. Compressive Strength

**Figure 4:**
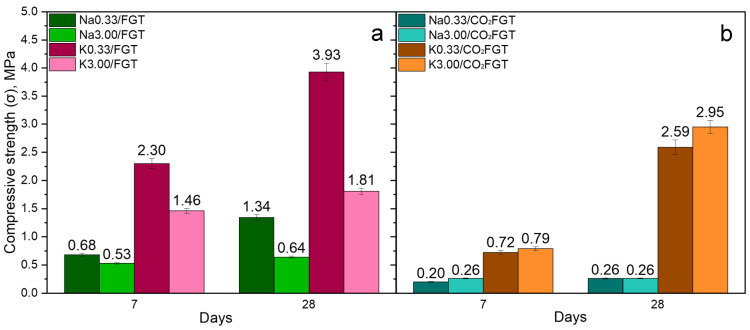
Compressive strength of FGT (**a**) and CO_2_FGT (**b**) fly ashes activated by potassium (K) or sodium (Na) alkali-activated materials.

To evaluate the mechanical properties of the obtained alkali-activated materials from carbonated (FGT) and uncarbonated (CO_2_FGT) fly ashes, the compressive strength values were examined after 7 and 28 days. After 28 days, the saturation in compressive strength value was obtained. The results are summarized in Figure 4. It can be clearly seen that fly ash mineral carbonation had a significant influence on the mechanical properties of the obtained alkali-activated materials. For uncarbonated fly ash alkali-activated materials (Figure 4a), the compressive strength values after 7 days were significantly higher (nearly 3-fold) than those obtained from carbonated fly ash (Figure 4b). Such a phenomenon might be a result of carbonate formation, which affects mechanical properties during alkali activation [46]. After 28 days, this trend was generally maintained, and the values obtained for uncarbonated activated materials were higher (except for the sample K3.00/CO_2_FGT). The differences can also be seen when comparing the type of activation agent used. For all examined series, the fly ashes activated using 8 mol dm^−3^ KOH and potassium silicate were characterized by higher compressive strength when compared with those obtained using 8 mol dm^−3^ NaOH and sodium silicate. In the research of [57], it can be found that a hydroxide concentration of 8 mol dm^−3^ is optimal to obtain sufficient compressive strength of the activated material. Moreover, higher compressive strength values were obtained after the application of potassium hydroxide than sodium hydroxide. In addition, ref. [58] reported that K^+^ ions are responsible for the condensation of the material, forming denser structures than those obtained during activation in the presence of Na^+^ ions. Such observation was also reported by [47]. It can also be a result of a smaller radius of Na^+^ than K^+^ ions. Hence, Na–O bonds are stronger than K–O bonds, which can, in turn, negatively affect the compressive strength of sodium-alkali-activated materials. Moreover, potassium alkali-activated materials were reported to exhibit more stable phase formation during thermal treatment, also reflected in lower mass loss and materials shrinkage compared with sodium alkali activation [27,47].

After 28 days, no significant difference was observed for carbonated fly ash activated using sodium alkali activation agents. On the other hand, materials obtained from uncarbonated fly ash (FGT) were characterized by improved compressive strength values for both potassium and sodium alkali activation agent treatments after 7 and 28 days. The highest compressive strength among the samples obtained was for K0.33/FGT, which was 3.93 MPa after 28 days. For carbonated fly ash, activation using potassium activation agents improved the compressive strength after 28 days more significantly than that for uncarbonated fly ash. In addition, the effect of the relative activation agent ratio (hydroxide:silicate) of 3.00 and 0.33 varied between the uncarbonated (FGT) and carbonated (CO_2_FGT) series. It is well known that, generally, a higher dose of Si (lower hydroxide:silicate ratio) contributes significantly to compressive strength [59]. However, it can be seen that after mineral carbonation, a higher dose of hydroxide favors an increase in compressive strength. The opposite trend was observed for uncarbonated fly ash, in which higher compressive strength values were obtained when the hydroxide:silicate ratio was 0.33. It has been reported that a greater dose of hydroxide accelerates the interaction between the Na^+^ ions and CO_2_, forming carbonates, which can lower compressive strength [60]. In fact, such a trend was observed in the present study after both 7 and 28 days. The possible formation of carbonates (CO_2_ from fly ash) with Na^+^ and K^+^ ions can, in fact, affect the compressive strength of the materials after 7 days. It might be assumed that the formation of carbonates is more favored for sodium than for potassium, for which samples the compressive strength values after both 7 and 28 days were higher. As was reported by Georget et al. [61], due to so-called alkali leaching, alkali metal ions (Na^+^ and K^+^) are highly mobile and can be transferred from the pores to the surface of the material, while mineral carbonation occurs. In addition, Ye et al. [62] reported that carbonated sodium-alkali-activated slags exhibited higher mass loss as a result of greater dehydration at low temperatures (30–250 °C) than their potassium counterparts. Hence, as a result of higher dehydration occurring for sodium-alkali-activated materials (both carbonated and uncarbonated), the compressive strength was found to be lower than for potassium-alkali-activated counterparts. A high content of calcium and chlorine within the fly ashes can also lower the compressive strength of the obtained alkali-activated materials by affecting the formation of the aluminosilicate network [46]. In the research by Yan et al. [63], it can be found that MSWI fly ashes were also investigated as additives for geopolymer synthesis. Moreover, the addition of 25% MSWI fly ash to cement caused a significant drop in the compressive strength of the obtained materials (<4 MPa after 28 days). What is noteworthy is that in this study, the solid phase used for alkali activation consists of MSWI fly ash only.

### 3.4. Future Perspectives

One of the major challenges for the cement industry is the constant search for a replacement for ordinary Portland cement (OPC). OPC, despite being used worldwide, exhibits a loss in mechanical strength during exposure to elevated temperatures (fire), which may cause a potential risk. In addition, the production of OPC is responsible for high CO_2_ emissions to the atmosphere and high energy consumption (preparation of raw material at about 1450 °C) [64,65]. Moreover, emissions of CO_2_ (which account for about 3.7 billion tons) originate from the utilization of carbonated waste construction materials. Hence, there is a constant need to search for alternative materials for the building industry [34]. Geopolymers were found to possess high compressive strengths (>40 MPa) with simultaneous resistance toward fire (small drop in compressive strength). The challenges in geopolymer production involve finding suitable raw materials characterized by a sufficient Si/Al ratio, activation procedure, and scalability of their production. An additional eco-friendly aspect of geopolymer production is the utility of wastes (fly ashes, slags) as raw materials and alkali activation agents (hydroxides, silicates) as the key reactants [66,67]. In fact, there are limited studies regarding the utilization of HCFA, which is a more problematic energetic waste. For this reason, there are works considering the small addition of HCFA (about 20%) to geopolymer synthesis with other raw materials, and high compressive strength values (>40 MPa) were reported [68]. A novel approach is the concept of so-called “intelligent buildings”, where materials used in the building industry can remove CO_2_ from the air [69,70].

The environmental aspects of geopolymer production, which should be taken into account while upscaling their production, involve the impact of alkali activation waste utilization, energy consumption, and water consumption [71]. A reduction in the impact of alkali activation solution on the environment can be achieved through optimization of the dose and concentration of hydroxides and silicates [72]. Notably, the wastewater solution reached in OH^−^ ions (high pH) and Al^3+^ and Si^4+^ ions dissolved forms can serve as raw material for the production of value-added materials such as zeolites in the hydrothermal process [10].

## 4. Conclusions

MSWI fly ash was carbonated using a static method aiming to investigate CO_2_ sequestration by waste material. An emphasis was given to the opportunity to store CO_2_ in such waste and to the effect of alkali activation on the compressive strength of obtained materials. The ability to store CO_2_ by the MSWI fly ash was 0.25 mmol CO_2_ g^−1^. Such value remains comparable with the literature and brings a valuable opportunity to decarbonize the energy sector in situ. A novel approach was the determination of the mechanical properties (compressive strength of alkali-activated fly ashes both prior to and after mineral carbonation). A series of alkali-activated municipal solid waste incineration fly ashes were synthesized. The obtained materials were characterized by compressive strength comparable to those obtained in the literature for this group of raw materials. The selected activator type (potassium or sodium) had a significant effect on the results. Potassium hydroxide mixed with potassium silicate allowed to produce materials characterized by compressive strengths of 3.93 and 2.59 MPa after 28 days for uncarbonated and carbonated fly ashes, respectively. Due to the high content of free CaO (about 16%), the application of MSWI fly ash directly for the production of cement would be limited in light of the European Union standards. It should also be stated that the treatment of MSWI fly ash containing high alkali earth metals with simultaneous low content of Al would lead to a significant drop in the compressive strength, as the geopolymerization process is limited. In addition, the term “geopolymer” should be used with caution in terms of such materials. However, problematic wastes can become additives for geopolymer synthesis. Moreover, the ability to stabilize CO_2_ as well as heavy metals within alkali-activated materials brings an opportunity to lower their emissions to the environment, which is in line with the global policy of environmental protection.

## Figures and Tables

**Table 1:** Sample description.

Sample	Used Alkali Activation Agent (Hydroxide:Silicate)	Alkali Activation Agent Ratio (Hydroxide:Silicate)
K3.00/FGT	8 mol dm^−3^ KOH:potassium silicate	3:1
K0.33/FGT	8 mol dm^−3^ KOH:potassium silicate	1:3
Na3.00/FGT	8 mol dm^−3^ NaOH:sodium silicate	3:1
Na0.33/FGT	8 mol dm^−3^ NaOH:sodium silicate	1:3
K3.00/CO_2_FGT	8 mol dm^−3^ KOH:potassium silicate	3:1
K0.33/CO_2_FGT	8 mol dm^−3^ KOH:potassium silicate	1:3
Na3.00/CO_2_FGT	8 mol dm^−3^ NaOH:sodium silicate	3:1
Na0.33/CO_2_FGT	8 mol dm^−3^ NaOH:sodium silicate	1:3

**Table 2:** Chemical composition of investigated fly ashes and their alkali-activated derivatives.

Sample	Component, wt.%											
	CaO	Cl	SO_3_	K_2_O	Na_2_O	ZnO	SiO_2_	Fe_2_O_3_	TiO_2_	As_2_O_3_	PbO	Br
Fly ashes												
FGT	51.54	18.65	16.77	4.45	3.71	1.66	1.07	0.89	0.60	0.34	0.18	0.14
CO_2_FGT	52.87	19.03	15.26	4.42	3.76	1.71	0.91	0.90	0.49	0.32	0.19	0.14
FGT-derived alkali-activated materials												
K3.00/FGT	53.00	13.44	13.99	7.77	3.49	1.71	4.68	0.79	0.47	0.33	0.18	0.15
K0.33/FGT	53.99	13.75	10.24	11.42	3.61	0.78	5.41	0.48	0.32	-	-	-
Na3.00/FGT	55.13	12.44	12.40	4.12	7.35	1.96	4.40	0.82	0.71	0.32	0.17	0.18
Na0.33/FGT	51.46	12.50	12.91	3.63	10.75	1.58	5.23	0.84	0.66	0.31	0.13	-
CO_2_FGT-derived alkali-activated materials												
K3.00/CO_2_FGT	53.54	15.23	12.30	7.47	3.64	0.96	5.83	0.53	0.28	-	0.12	0.10
K0.33/CO_2_FGT	53.81	12.60	12.56	8.11	3.53	1.16	6.79	0.81	0.63	-	-	-
Na3.00/CO_2_FGT	53.19	13.24	13.59	3.64	7.52	1.13	5.50	0.84	0.60	0.36	0.19	0.20
Na0.33/CO_2_FGT	52.44	11.47	12.01	3.47	10.51	1.86	6.48	0.81	0.74	-	-	0.21

## Data Availability

Data is contained within the article or Supplementary Materials.

## Supplementary Materials

The following supporting information can be downloaded at: https://www.mdpi.com/article/10.3390/ma16186094/s1, Table S1: FTIR raw data.

## References

[1] Suraneni P., Burris L., Shearer C.R., Hooton R.D. (2021). ASTM C618 Fly Ash Specification: Comparison with Other Specifications, Shortcomings, and Solutions. ACI Mater. J..

[2] Dindi A., Quang D.V., Vega L.F., Nashef E., Abu-Zahra M.R.M. (2019). Applications of Fly Ash for CO_2_ Capture, Utilization, and Storage. J. CO2 Util..

[3] Verma C., Madan S., Hussain A. (2016). Heavy Metal Contamination of Groundwater Due to Fly Ash Disposal of Coal-Fired Thermal Power Plant, Parichha, Jhansi, India. Cogent Eng..

[4] Mishra M., Sahu S.K., Mangaraj P., Beig G. (2023). Assessment of Hazardous Radionuclide Emission Due to Fly Ash from Fossil Fuel Combustion in Industrial Activities in India and Its Impact on Public. J. Environ. Manag..

[5] Grabias-Blicharz E., Franus W. (2023). A Critical Review on Mechanochemical Processing of Fly Ash and Fly Ash-Derived Materials. Sci. Total Environ..

[6] Fly Ash Market Size USD 19.59 Billion by 2030. https://www.vantagemarketresearch.com/industry-report/fly-ash-market-1875.

[7] Luo Y., Brouwers H.J.H., Yu Q. (2023). Understanding the Gel Compatibility and Thermal Behavior of Alkali Activated Class F Fly Ash/Ladle Slag: The Underlying Role of Ca Availability. Cem. Concr. Res..

[8] Islam M.S., Elahi T.E., Shahriar A.R., Mumtaz N. (2020). Effectiveness of Fly Ash and Cement for Compressed Stabilized Earth Block Construction. Constr. Build. Mater..

[9] Mokrzycki J., Fedyna M., Marzec M., Panek R., Szerement J., Marcińska-Mazur L., Jarosz R., Bajda T., Franus W., Mierzwa-Hersztek M. (2022). The Influence of Zeolite X Ion-Exchangeable Forms and Impregnation with Copper Nitrate on the Adsorption of Phosphate Ions from Aqueous Solutions. J. Water Process Eng..

[10] Mokrzycki J., Franus W., Panek R., Sobczyk M., Rusiniak P., Szerement J., Jarosz R., Marcińska-Mazur L., Bajda T., Mierzwa-Hersztek M. (2023). Zeolite Composite Materials from Fly Ash: An Assessment of Physicochemical and Adsorption Properties. Materials.

[11] Mokrzycki J., Fedyna M., Marzec M., Szerement J., Panek R., Klimek A., Bajda T., Mierzwa-Hersztek M. (2022). Copper Ion-Exchanged Zeolite X from Fly Ash as an Efficient Adsorbent of Phosphate Ions from Aqueous Solutions. J. Environ. Chem. Eng..

[12] Zhang H., Gan S., Sun H., Yang H., Xie S. (2022). Fly-Ash-Based Hierarchical MCM-41 Molecular Sieve as an Efficient Adsorbent for Methylene Blue Removal from Wastewater over a Wide PH. ChemistrySelect.

[13] Czarna-Juszkiewicz D., Cader J., Wdowin M. (2020). From Coal Ashes to Solid Sorbents for Hydrogen Storage. J. Clean. Prod..

[14] Zarębska K., Zabierowski P., Gazda-Grzywacz M., Czuma N., Baran P. (2022). Fly Ash-Based Geopolymers with Refractoriness Properties. Clean. Technol. Environ. Policy.

[15] Baran P., Nazarko M., Włosińska E., Kanciruk A., Zarębska K. (2021). Synthesis of Geopolymers Derived from Fly Ash with an Addition of Perlite. J. Clean. Prod..

[16] Singh N.B. (2018). Fly Ash-Based Geopolymer Binder: A Future Construction Material. Minerals.

[17] Davidovits J. (1989). Geopolymers and Geopolymeric Materials. J. Therm. Anal..

[18] Khale D., Chaudhary R. (2007). Mechanism of Geopolymerization and Factors Influencing Its Development: A Review. J. Mater. Sci..

[19] Zhang P., Gao Z., Wang J., Guo J., Hu S., Ling Y. (2020). Properties of Fresh and Hardened Fly Ash/Slag Based Geopolymer Concrete: A Review. J. Clean. Prod..

[20] Colangelo F., Farina I., Travaglioni M., Salzano C., Cioffi R., Petrillo A. (2021). Eco-Efficient Industrial Waste Recycling for the Manufacturing of Fibre Reinforced Innovative Geopolymer Mortars: Integrated Waste Management and Green Product Development through LCA. J. Clean. Prod..

[21] Jaya N.A., Yun-Ming L., Cheng-Yong H., Abdullah M.M.A.B., Hussin K. (2020). Correlation between Pore Structure, Compressive Strength and Thermal Conductivity of Porous Metakaolin Geopolymer. Constr. Build. Mater..

[22] Wang Y., Liu X., Zhang W., Li Z., Zhang Y., Li Y., Ren Y. (2020). Effects of Si/Al Ratio on the Efflorescence and Properties of Fly Ash Based Geopolymer. J. Clean. Prod..

[23] Hamdane H., Tamraoui Y., Mansouri S., Oumam M., Bouih A., el Ghailassi T., Boulif R., Manoun B., Hannache H. (2020). Effect of Alkali-Mixed Content and Thermally Untreated Phosphate Sludge Dosages on Some Properties of Metakaolin Based Geopolymer Material. Mater. Chem. Phys..

[24] Xu Z., Yue J., Pang G., Li R., Zhang P., Xu S. (2021). Influence of the Activator Concentration and Solid/Liquid Ratio on the Strength and Shrinkage Characteristics of Alkali-Activated Slag Geopolymer Pastes. Adv. Civ. Eng..

[25] Lin H., Liu H., Li Y., Kong X. (2021). Properties and Reaction Mechanism of Phosphoric Acid Activated Metakaolin Geopolymer at Varied Curing Temperatures. Cem. Concr. Res..

[26] Khalifeh M., Saasen A., Vralstad T., Hodne H. (2014). Potential Utilization of Class C Fly Ash-Based Geopolymer in Oil Well Cementing Operations. Cem. Concr. Compos..

[27] Klima K.M., Schollbach K., Brouwers H.J.H., Yu Q. (2022). Enhancing the Thermal Performance of Class F Fly Ash-Based Geopolymer by Sodalite. Constr. Build. Mater..

[28] Kan L., Shi R., Zhao Y., Duan X., Wu M. (2020). Feasibility Study on Using Incineration Fly Ash from Municipal Solid Waste to Develop High Ductile Alkali-Activated Composites. J. Clean. Prod..

[29] Zarębska K., Szczurowski J., Gazda-Grzywacz M., Wróbel W., Bator J., Baran P. (2023). Geopolymer Building Materials Based on Fly Ash in Terms of Removing SO_2_, CO_2_, and Water Vapor. Energies.

[30] Beaino S., El Hage P., Sonnier R., Seif S., El Hage R. (2022). Novel Foaming-Agent Free Insulating Geopolymer Based on Industrial Fly Ash and Rice Husk. Molecules.

[31] Reynolds B., Reddy K.J., Argyle M.D. (2014). Field Application of Accelerated Mineral Carbonation. Minerals.

[32] Chen J., Lin X., Li M., Mao T., Li X., Yan J. (2022). Heavy Metal Solidification and CO_2_ Sequestration from MSWI Fly Ash by Calcium Carbonate Oligomer Regulation. J. Clean. Prod..

[33] Rackley S.A. (2017). Mineral Carbonation. Carbon Capture and Storage.

[34] Liu W., Teng L., Rohani S., Qin Z., Zhao B., Xu C.C., Ren S., Liu Q., Liang B. (2021). CO_2_ Mineral Carbonation Using Industrial Solid Wastes: A Review of Recent Developments. Chem. Eng. J..

[35] Tamilselvi Dananjayan R.R., Kandasamy P., Andimuthu R. (2016). Direct Mineral Carbonation of Coal Fly Ash for CO_2_ Sequestration. J. Clean. Prod..

[36] Rausis K., Ćwik A., Casanova I., Zarębska K. (2021). Carbonation of High-Ca Fly Ashes under Flue Gas Conditions: Implications for Their Valorization in the Construction Industry. Crystals.

[37] Sobala J., Czuma N., Bator J., Baran P., Zabierowski P., Zarębska K. (2020). Scholars International Journal of Chemistry and Material Sciences the Mineral Carbonation Process of High Calcium Fly Ash under Elevated Carbon Dioxide Pressure and Temperature. Sch. Int. J. Chem. Mater. Sci..

[38] PN-EN 451-1:2017-06-Wersja Angielska. https://sklep.pkn.pl/pn-en-451-1-2017-06e.html.

[39] PN-EN 196-1:2016-07-Wersja Polska. https://sklep.pkn.pl/pn-en-196-1-2016-07p.html.

[40] Liu W., Su S., Xu K., Chen Q., Xu J., Sun Z., Wang Y., Hu S., Wang X., Xue Y. (2018). CO_2_ Sequestration by Direct Gas–Solid Carbonation of Fly Ash with Steam Addition. J. Clean. Prod..

[41] Siriruang C., Toochinda P., Julnipitawong P., Tangtermsirikul S. (2016). CO_2_ Capture Using Fly Ash from Coal Fired Power Plant and Applications of CO2-Captured Fly Ash as a Mineral Admixture for Concrete. J. Environ. Manag..

[42] Gupta S., Kashani A., Mahmood A.H., Han T. (2021). Carbon Sequestration in Cementitious Composites Using Biochar and Fly Ash–Effect on Mechanical and Durability Properties. Constr. Build. Mater..

[43] Miao E., Du Y., Zheng X., Zhang X., Xiong Z., Zhao Y., Zhang J. (2023). Kinetic Analysis on CO2 Sequestration from Flue Gas through Direct Aqueous Mineral Carbonation of Circulating Fluidized Bed Combustion Fly Ash. Fuel.

[44] Zhu M., Li Q., Liang C., Ma Z., Liu Z. (2022). Mechanistic Investigation for Solidification of Pb in Fly Ash by Alkali Mineral Slag&mdash;Calcium Chloroaluminate as an Example. Minerals.

[45] Wu K., Shi H., De Schutter G., Guo X., Ye G. (2012). Experimental Study on Alinite Ecocement Clinker Preparation from Municipal Solid Waste Incineration Fly Ash. Mater. Struct./Mater. Constr..

[46] Zheng L., Wang C., Wang W., Shi Y., Gao X. (2011). Immobilization of MSWI Fly Ash through Geopolymerization: Effects of Water-Wash. Waste Manag..

[47] Klima K.M., Schollbach K., Brouwers H.J.H., Yu Q. (2022). Thermal and Fire Resistance of Class F Fly Ash Based Geopolymers—A Review. Constr. Build. Mater..

[48] Liu T., Li S., Chen Y., Brouwers H.J.H., Yu Q. (2023). In-Situ Formation of Layered Double Hydroxides in MgO–NaAlO2-Activated GGBS / MSWI BA: Impact of Mg2+ on Reaction Mechanism and Leaching Behavior. Cem. Concr. Compos..

[49] Tome S., Etoh M.A., Etame J., Kumar S. (2020). Improved Reactivity of Volcanic Ash Using Municipal Solid Incinerator Fly Ash for Alkali-Activated Cement Synthesis. Waste Biomass Valorization.

[50] Marieta C., Guerrero A., Leon I. (2021). Municipal Solid Waste Incineration Fly Ash to Produce Eco-Friendly Binders for Sustainable Building Construction. Waste Manag..

[51] (2012). Fly Ash for Concrete-Part 1: Definition, Specifications and Conformity Criteria.

[52] Ćwik A., Casanova I., Rausis K., Zarȩbska K. (2019). Utilization of High-Calcium Fly Ashes through Mineral Carbonation: The Cases for Greece, Poland and Spain. J. CO2 Util..

[53] Tian Q., Bai Y., Pan Y., Chen C., Yao S., Sasaki K., Zhang H. (2022). Application of Geopolymer in Stabilization/Solidification of Hazardous Pollutants: A Review. Molecules.

[54] Liu Z., Yang Y., Zhang Y., Yue Y., Zhang J., Qian G. (2022). Controlling Role of CaClOH in the Process of Dechlorination for Municipal Solid Incineration Fly Ash Utilization. ACS ES T Eng..

[55] Liu J., Hu L., Tang L., Ren J. (2021). Utilisation of Municipal Solid Waste Incinerator (MSWI) Fly Ash with Metakaolin for Preparation of Alkali-Activated Cementitious Material. J. Hazard. Mater..

[56] Wu W., Matalkah F., Darsanasiri A.G.N.D., Soroushian P. (2019). Fluidized Bed Combustion Coal Fly Ash: Comparative Evaluation for Potential Use in Alkali-Activated Binders. Int. J. Coal Prep. Util..

[57] Rehman S.K.U., Imtiaz L., Aslam F., Khan M.K., Haseeb M., Javed M.F., Alyousef R., Alabduljabbar H. (2020). Experimental Investigation of NaOH and KOH Mixture in SCBA-Based Geopolymer Cement Composite. Materials.

[58] El Alouani M., Alehyen S., El Achouri M., Hajjaji A., Ennawaoui C., Taibi M. (2020). Influence of the Nature and Rate of Alkaline Activator on the Physicochemical Properties of Fly Ash-Based Geopolymers. Adv. Civ. Eng..

[59] Prasanphan S., Hemra K., Wannagon A., Kobayashi T., Onutai S., Jiemsirilers S. (2023). 29Si and 27Al NMR Study of the Structural Transformation of Calcined Kaolin Residue-Based Geopolymer Using Low Alkali Activator Content for Sustainable Construction Materials. J. Build. Eng..

[60] Zhao S., Muhammad F., Yu L., Xia M., Huang X., Jiao B., Lu N., Li D. (2019). Solidification/Stabilization of Municipal Solid Waste Incineration Fly Ash Using Uncalcined Coal Gangue–Based Alkali-Activated Cementitious Materials. Environ. Sci. Pollut. Res..

[61] Georget F., Soja W., Scrivener K.L. (2020). Characteristic Lengths of the Carbonation Front in Naturally Carbonated Cement Pastes: Implications for Reactive Transport Models. Cem. Concr. Res..

[62] Ye H., Cai R., Tian Z. (2020). Natural Carbonation-Induced Phase and Molecular Evolution of Alkali-Activated Slag: Effect of Activator Composition and Curing Temperature. Constr. Build. Mater..

[63] Yan K., Gao F., Sun H., Ge D., Yang S. (2019). Effects of Municipal Solid Waste Incineration Fly Ash on the Characterization of Cement-Stabilized Macadam. Constr. Build. Mater..

[64] Vijaya Prasad B., Paul Daniel A.P., Anand N., Yadav S.K. (2022). Strength and Microstructure Behaviour of High Calcium Fly Ash Based Sustainable Geo Polymer Concrete. J. Eng. Des. Technol..

[65] Wong L.S. (2022). Durability Performance of Geopolymer Concrete: A Review. Polymers.

[66] Danish A., Ozbakkaloglu T., Ali Mosaberpanah M., Salim M.U., Bayram M., Yeon J.H., Jafar K. (2022). Sustainability Benefits and Commercialization Challenges and Strategies of Geopolymer Concrete: A Review. J. Build. Eng..

[67] Alhawat M., Ashour A., Yildirim G., Aldemir A., Sahmaran M. (2022). Properties of Geopolymers Sourced from Construction and Demolition Waste: A Review. J. Build. Eng..

[68] Prabha V.C., Revathi V., Sivamurthy Reddy S. (2021). Ambient Cured High Calcium Fly Ash Geopolymer Concrete with Dolomite Powder. Eur. J. Environ. Civ. Eng..

[69] Freire A.L., Moura-Nickel C.D., Scaratti G., De Rossi A., Araújo M.H., De Noni Júnior A., Rodrigues A.E., Castellón E.R., de Fátima Peralta Muniz Moreira R. (2020). Geopolymers Produced with Fly Ash and Rice Husk Ash Applied to CO2 Capture. J. Clean. Prod..

[70] Han L., Wang X., Wu B., Zhu S., Wang J., Zhang Y. (2022). In-Situ Synthesis of Zeolite X in Foam Geopolymer as a CO2 Adsorbent. J. Clean. Prod..

[71] Bajpai R., Choudhary K., Srivastava A., Sangwan K.S., Singh M. (2020). Environmental Impact Assessment of Fly Ash and Silica Fume Based Geopolymer Concrete. J. Clean. Prod..

[72] Meshram R.B., Kumar S. (2022). Comparative Life Cycle Assessment (LCA) of Geopolymer Cement Manufacturing with Portland Cement in Indian Context. Int. J. Environ. Sci. Technol..

